# Pantoprazole impairs fracture healing in aged mice

**DOI:** 10.1038/s41598-020-79605-3

**Published:** 2020-12-23

**Authors:** Maximilian M. Menger, Philipp Bremer, Claudia Scheuer, Mika F. Rollmann, Benedikt J. Braun, Steven C. Herath, Marcel Orth, Thomas Später, Tim Pohlemann, Michael D. Menger, Tina Histing

**Affiliations:** 1 Institute for Clinical & Experimental Surgery, Saarland University, 66421 Homburg/Saar, Germany; 2 Department of Trauma and Reconstructive Surgery, BG Trauma Center Tuebingen, Eberhard Karls University Tuebingen, 72076 Tuebingen, Germany; 3 Department of Trauma, Hand and Reconstructive Surgery, Saarland University, 66421 Homburg/Saar, Germany

**Keywords:** Health care, Medical research, Pathogenesis

## Abstract

Proton pump inhibitors (PPIs) belong to the most common medication in geriatric medicine. They are known to reduce osteoclast activity and to delay fracture healing in young adult mice. Because differentiation and proliferation in fracture healing as well as pharmacologic actions of drugs markedly differ in the elderly compared to the young, we herein studied the effect of the PPI pantoprazole on bone healing in aged mice using a murine fracture model. Bone healing was analyzed by biomechanical, histomorphometric, radiological and protein biochemical analyses. The biomechanical analysis revealed a significantly reduced bending stiffness in pantoprazole-treated animals when compared to controls. This was associated with a decreased amount of bone tissue within the callus, a reduced trabecular thickness and a higher amount of fibrous tissue. Furthermore, the number of osteoclasts in pantoprazole-treated animals was significantly increased at 2 weeks and decreased at 5 weeks after fracture, indicating an acceleration of bone turnover. Western blot analysis showed a lower expression of the bone morphogenetic protein-4 (BMP-4), whereas the expression of the pro-angiogenic parameters was higher when compared to controls. Thus, pantoprazole impairs fracture healing in aged mice by affecting angiogenic and osteogenic growth factor expression, osteoclast activity and bone formation.

## Introduction

The population of the elderly will considerably increase in the next decades, making the treatment of geriatric patients a major health issue^1^. This population suffers from a higher risk of bone fractures, which is associated with an elevated mortality and a reduced healing potential^2^. Simultaneously, the use of proton pump inhibitors (PPIs) among the elderly has markedly increased during the last decades, becoming one of the most widely prescribed medications in geriatric medicine^3^. These potent acid-suppressing agents are commonly used for the therapy of reflux esophagitis as well as peptic and other gastrointestinal disorders^4,5^. Furthermore, PPIs are used in combination with analgesic drugs, especially with nonsteroidal anti-inflammatory drugs (NSAIDs). They should prevent stress ulcers by inhibiting the gastric proton-pump (H^+^/K^+^-ATPase) activity in parietal cells during basal and stimulated gastric acid secretion^6,7^.

Despite the symptomatic benefits of PPIs, it is well known that chronic acid suppression can have also detrimental physiological effects. PPIs inhibit calcium and vitamin B_12_ absorption, resulting in an increased rate of bone loss, a low bone mineral density (BMD) and, therefore, a greater risk for fractures^8–10^. Epidemiological studies could verify that PPI therapy in the elderly is associated with an increased risk of non-spine fractures^11^, in particular hip^12–14^ and other osteoporosis-related fractures^15–17^. In addition, we demonstrated in a previous study using a murine femur fracture model that in young adult mice PPI administration delays fracture healing^18^.

However, there is also evidence that PPIs can prevent bone loss by inhibiting the osteoclastic vacuolar H^+^-ATPase (V-ATPase)^19,20^. This enzyme is responsible for creating an acidic environment between the ruffled border of osteoclasts and the bone tissue. Thereby, lytic enzymes are activated at the bone-apposed plasma membrane of the osteoclast and bone is resorbed within the process of remodeling^21^. Interestingly, Visentin et al.^22^ could show that a selective inhibitor of the V-ATPase protects from bone loss in ovariectomized rats. Moreover, recent studies implicate an altered activity of the V-ATPase in cellular aging and longevity^23^. Hence, the inhibition of the V-ATPase by PPIs may have a different effect in aged individuals when compared to the young and may therefore, prevent osteoporosis and, more importantly, benefit bone healing in the elderly.

On the other hand, fracture healing in geriatric patients is subjected to significant alterations including a decreased differentiation and proliferation of stem cells^24,25^ as well as the delay of chondrogenesis and osteochondral ossification^26^. Moreover, aging involves a progressive decline in the functional reserve of multiple organs, such as hepatic clearance and renal extraction^27^. These physiological changes do not only affect drug pharmacokinetics and metabolism, but may also alter the mode of action of specific drugs, including PPIs. Of interest, there is a complete lack of information about the effects of PPIs on bone healing and regeneration in the elderly. Herein, we hypothesized that PPIs affect fracture healing also in aged mice. To prove this hypothesis, we studied in aged mice the effects of pantoprazole on fracture healing using a standardized femur fracture model.

## Results

In CD-1 mice transverse midshaft femur fractures were induced by a 3-point bending device. The fractures were stabilized using intramedullary medical stainless steel screws. Animals were treated daily by intraperitoneal (i.p.) injection of 100 mg/kg body weight (BW) pantoprazole (Nycomed, Konstanz, Germany). Control animals received the same amounts of vehicle (saline). Animals were studied directly after surgery and at 2 weeks or 5 weeks after fracture healing.

### Radiological analysis

**Figure 1:**
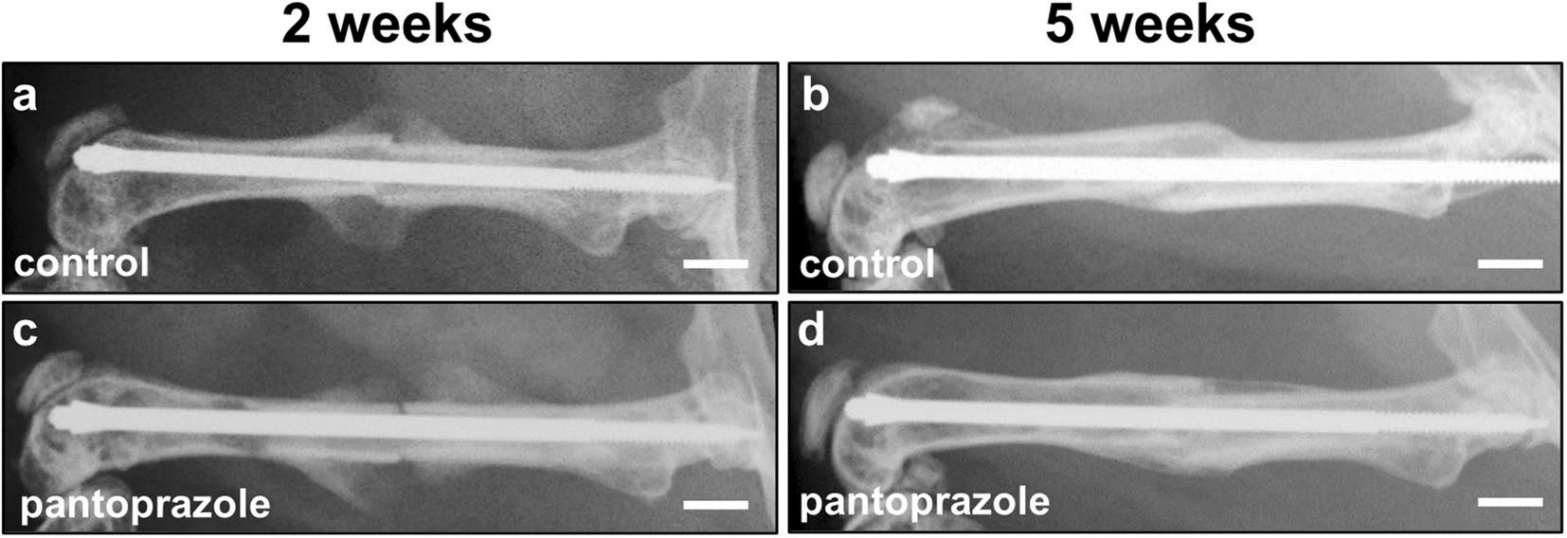
Radiological analysis of mice femora. Radiological images of the femora of controls (**a**,**b**) and pantoprazole-treated animals (**c**,**d**) at 2 weeks (**a**,**c**) and 5 weeks (**b**,**d**) after fracture healing. Scale bars: 2 mm.

The radiography performed directly after surgery confirmed transverse midshaft femur fractures. It also confirmed the adequate positioning of the intramedullary implants. Radiological analysis further revealed a lower Goldberg score in pantoprazole-treated animals compared to controls at both 2 (0.9 ± 0.2 vs. 1.3 ± 0.2; *p* = 0.110) and 5 weeks (1.8 ± 0.2 vs. 2.0 ± 0.0; *p* = 0.201) after fracture healing (Fig. 1). However, this difference did not prove to be statistically significant.

### Biomechanical analysis

**Figure 2:**
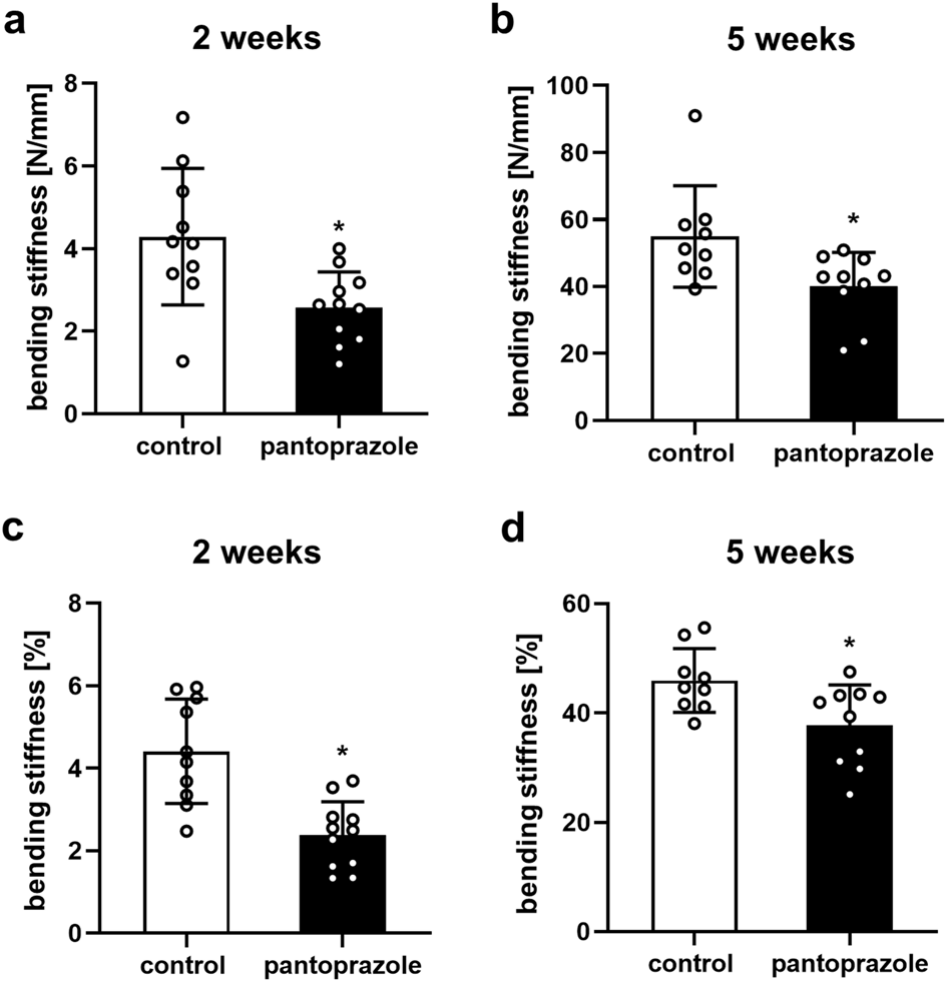
Biomechanical analysis of mice femora. Biomechanical analysis of bending stiffness 2 weeks (**a**,**c**) and 5 weeks (**b**,**d**) after fracture healing in controls (white bars) and pantoprazole-treated animals (black bars). Data are given in absolute values [N/mm] (**a**,**b**) and in percent to the contralateral, non-fractured femora [%] (**c**,**d**). Means ± SD; n = 9–12; *p < 0.05 vs. control.

Biomechanical analysis of the femora using a three-point bending device (Mini-Zwick Z 2.5; Zwick, Ulm, Germany) showed a significantly lower bending stiffness in the pantoprazole-treated animals at 2 (Fig. 2a,c) and 5 weeks (Fig. 2b,d) after fracture healing when compared to vehicle-treated controls. This significantly lower bending stiffness in pantoprazole-treated animals was found when comparing the absolute bending stiffness data (Fig. 2a,b) (*p* = 0.007 at 2 weeks, *p* = 0.021 at 5 weeks) but also when comparing the relative data (Fig. 2c,d, referring to the non-fractured contralateral femora) (*p* ≤ 0.001 at 2 weeks, *p* = 0.017 at 5 weeks). Of note, pantoprazole treatment showed no effect on the bending stiffness of the non-fractured contralateral femora at 2 and 5 weeks after fracture healing when compared to controls (95.9 ± 7.2 vs. 108.9 ± 2.7 N/mm; *p* = 0.098 and 118.6 ± 7.4 vs. 106.2 ± 7.2 N/mm; *p* = 0.153).

### Histological analysis

**Figure 3:**
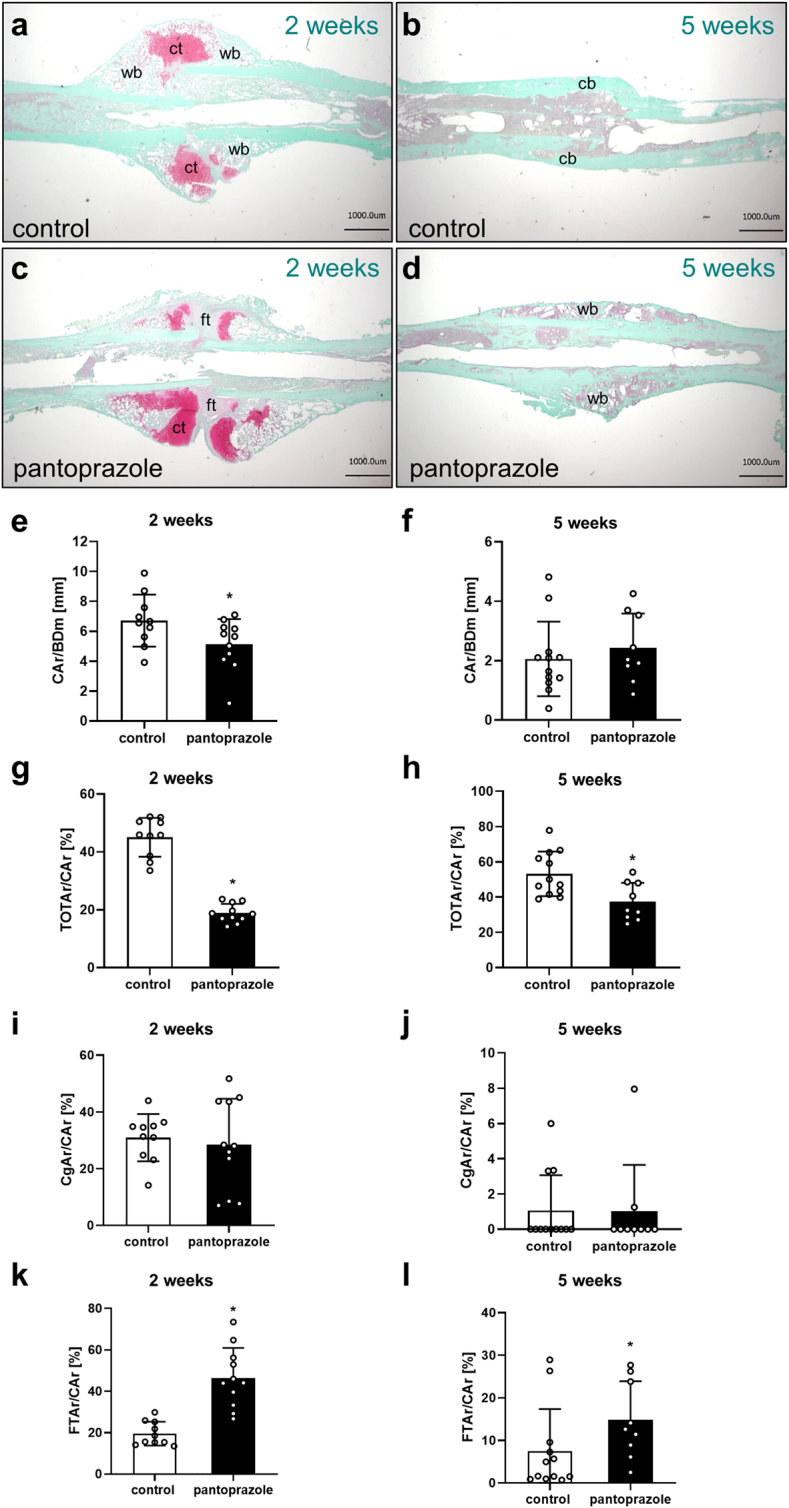
Histological and histomorphometrical analyses of mice femora. Representative histological images 2 weeks (**a**,**c**) and 5 weeks (**b**,**d**) after fracture healing in controls (**a**,**b**) and pantoprazole-treated animals (**c**,**d**). Fibrous tissue (ft), cartilage tissue (ct), woven bone (wb) and cortical bone (cb) are indicated. Scale bars: 1000 µm. (**e**,**f**) Histomorphometric analysis of the total callus area (*CAr*) in relation to the diameter of the femur (*BDm*) at 2 weeks (**e**) and 5 weeks (**f**) after fracture healing in control (white bars) and pantoprazole-treated animals (black bars). (**g**–**l**) Histomorphometric analysis of the tissue distribution within the callus, including total osseous tissue callus area/total callus area (*TOTAr/CAr*, [%]) (**g**,**h**), cartilaginous callus area/total callus area (*CgAr/CAr*, [%]) (**i**,**j**), and fibrous tissue callus area/total callus area (*FTAr/CAr*, [%]) (**k**,**l**) at 2 weeks (**g**,**i**,**k**) and 5 weeks (**h**,**j**,**l**) after fracture healing in controls (*white bars*) and pantoprazole-treated animals (*black bars*). Means ± SD; n = 9–12; *p < 0.05 vs. control.

The histological analysis revealed in both groups a typical callus formation of secondary fracture healing, including intramembranous and endochondral ossification (Fig. 3a–d). At 2 weeks after fracture healing the total callus area in relation to the femoral diameter (CAr/BDm) was significantly lower in pantoprazole-treated animals compared to controls (Fig. 3e,f) (*p* = 0.048; *p* = 0.495 at 5 weeks). The analysis of callus composition at 2 (*p* =  < 0.001) and 5 (*p* = 0.007) weeks after fracture healing showed a significantly lower amount of bone tissue in pantoprazole-treated animals (Fig. 3g,h), whereas the amount of remnant fibrous tissue was significantly higher when compared to controls (Fig. 3k,l) (*p* ≤ 0.001 at 2 weeks; *p* = 0.036 at 5 weeks). Of note, the amount of cartilaginous tissue did not significantly differ between the two experimental groups, neither at 2 (*p* = 0.699) nor at 5 weeks (*p* = 0.924) after fracture healing (Fig. 3i,j).

**Figure 4:**
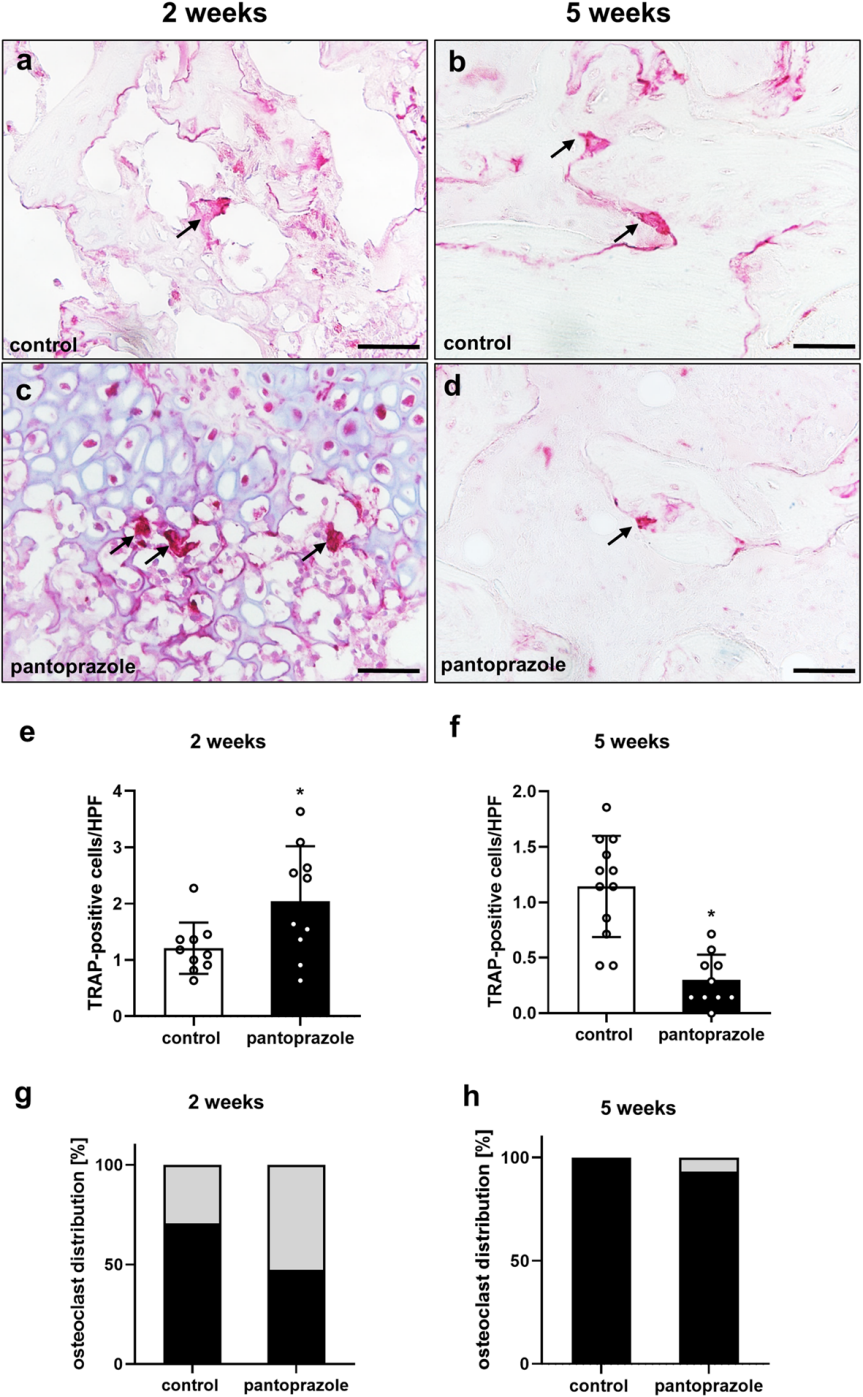
Histological analysis of osteoclastogenesis. (**a**–**d**) Representative histological sections of TRAP-positive osteoclasts (arrows) at 2 weeks (**a**,**c**) and 5 weeks (**b**,**d**) after fracture healing in controls and pantoprazole-treated animals. Scale bars: 50 µm. (**e**,**f**) Quantitative analysis of the number of TRAP-positive cells per high-power field (*HPF*) in the callus tissue at 2 weeks (**e**) and 5 weeks (**f**) of controls (white bars) and pantoprazole-treated animals (black bars). (**g**,**h**) Distribution of osteoclasts [%] within the osseous tissue (black) and cartilaginous tissue (gray) at 2 weeks (**g**) and 5 weeks (**h**) after fracture healing in controls and pantoprazole-treated animals. Means ± SD; n = 10–12; *p < 0.05 vs. control.

Furthermore, the number of tartrate-resistant acid phosphatase (TRAP)-positive osteoclasts within the callus tissue was also assessed by histology (Fig. 4a–d). The analysis showed a significantly enhanced number of osteoclasts at 2 weeks (*p* = 0.037) after fracture healing in pantoprazole-treated animals. In contrast, at 5 weeks (*p* ≤ 0.001) of pantoprazole treatment the number of osteoclasts was found significantly reduced when compared to vehicle-treated controls (Fig. 4e,f). Moreover, the distribution of the osteoclasts differed between the two study groups. In control animals after 2 weeks of fracture healing the majority of positively stained cells (~ 70%) were found in the osseous tissue. Only ~ 30% of the TRAP-positive osteoclasts were found in the cartilaginous tissue. In pantoprazole-treated mice after 2 weeks of fracture healing only ~ 45% of the TRAP-positive osteoclasts were found in the osseous tissue (*p* = 0.111), while the majority of the positive cells were found in the cartilaginous tissue (Fig. 4g) (*p* = 0.111). After 5 weeks in pantoprazole-treated animals all of the TRAP-positive cells were found in the osseous tissue (*p* = 0.493), while in controls a few positive cells were still found within the cartilaginous tissue (*p* = 0.129) of the callus (Fig. 4h).

**Figure 5:**
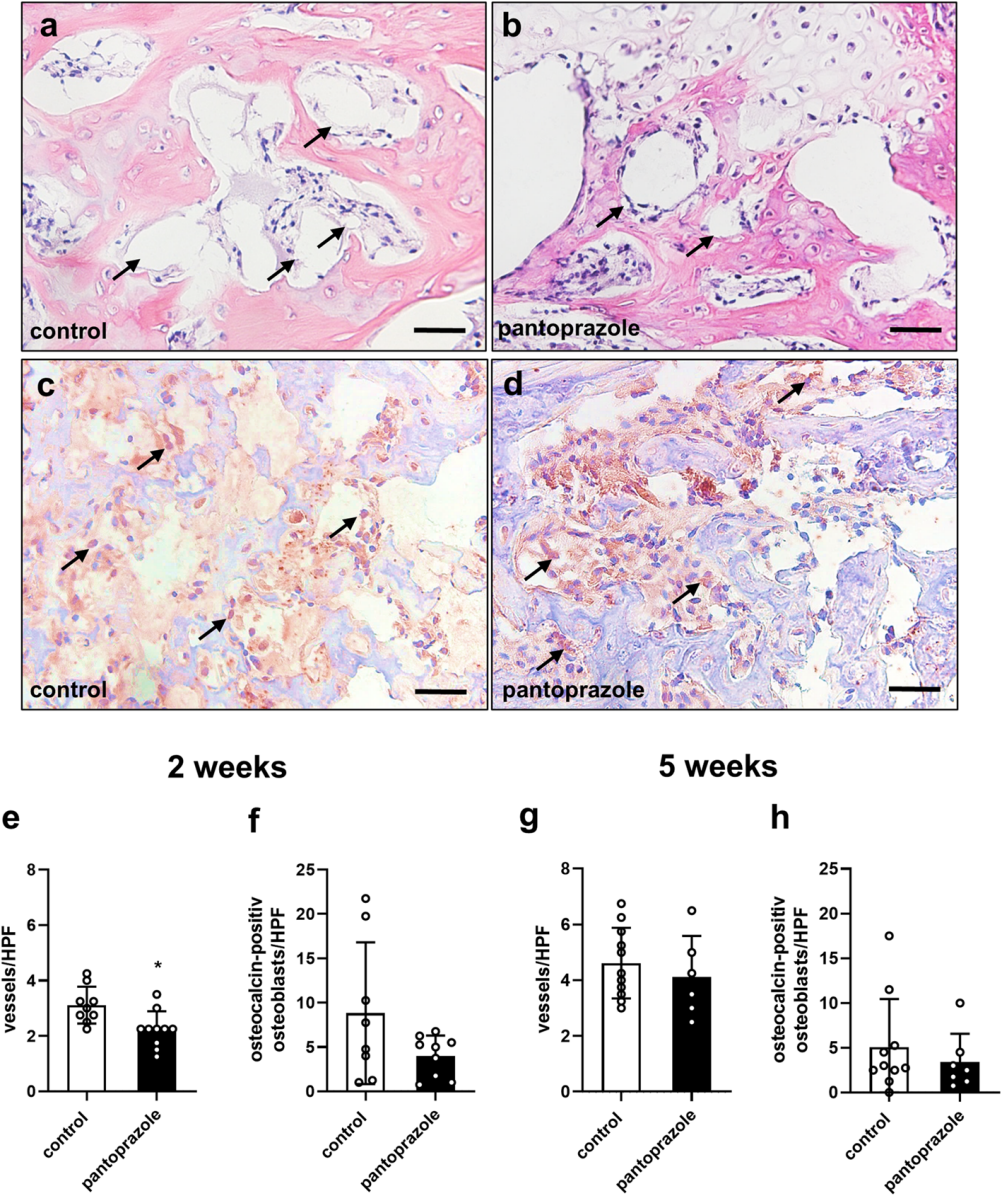
Histological analysis of vascularization and osteoblastogenesis. Representative histological sections of microvessels (arrows) at 2 weeks (**a**,**b**) and osteocalcin-positive osteoblasts at 2 weeks (**c**,**d**) after fracture healing in controls and pantoprazole-treated animal. Scale bars: 50 µm. Quantitative analysis of the number of microvessels (**e**,**g**) and osteocalcin-positive osteoblasts (**f**,**h**) per high-power field (*HPF*) in the callus tissue at 2 weeks (**e**,**f**) and 5 weeks (**g**,**h**) of controls (white bars) and pantoprazole-treated animals (black bars). Means ± SD; n = 6–11; *p < 0.05 vs. control.

Additional histological analysis revealed a significantly decreased vessel density in pantoprazole-treated animals at 2 weeks after fracture healing when compared to controls (Fig. 5a,b,e) (p = 0.010). At 5 weeks after fracture healing the vessel density of pantoprazole-treated animals did not significantly differ from that of controls (Fig. 5g) (p = 0.482).

Moreover, the number of osteoblasts within the callus tissue was examined by osteocalcin staining. This analysis revealed neither at 2 (Fig. 5c,d,f) nor at 5 weeks (Fig. 5h) after fracture healing a significant difference in the number of osteoblasts between the two study groups.

### µ-CT analysis

**Figure 6:**
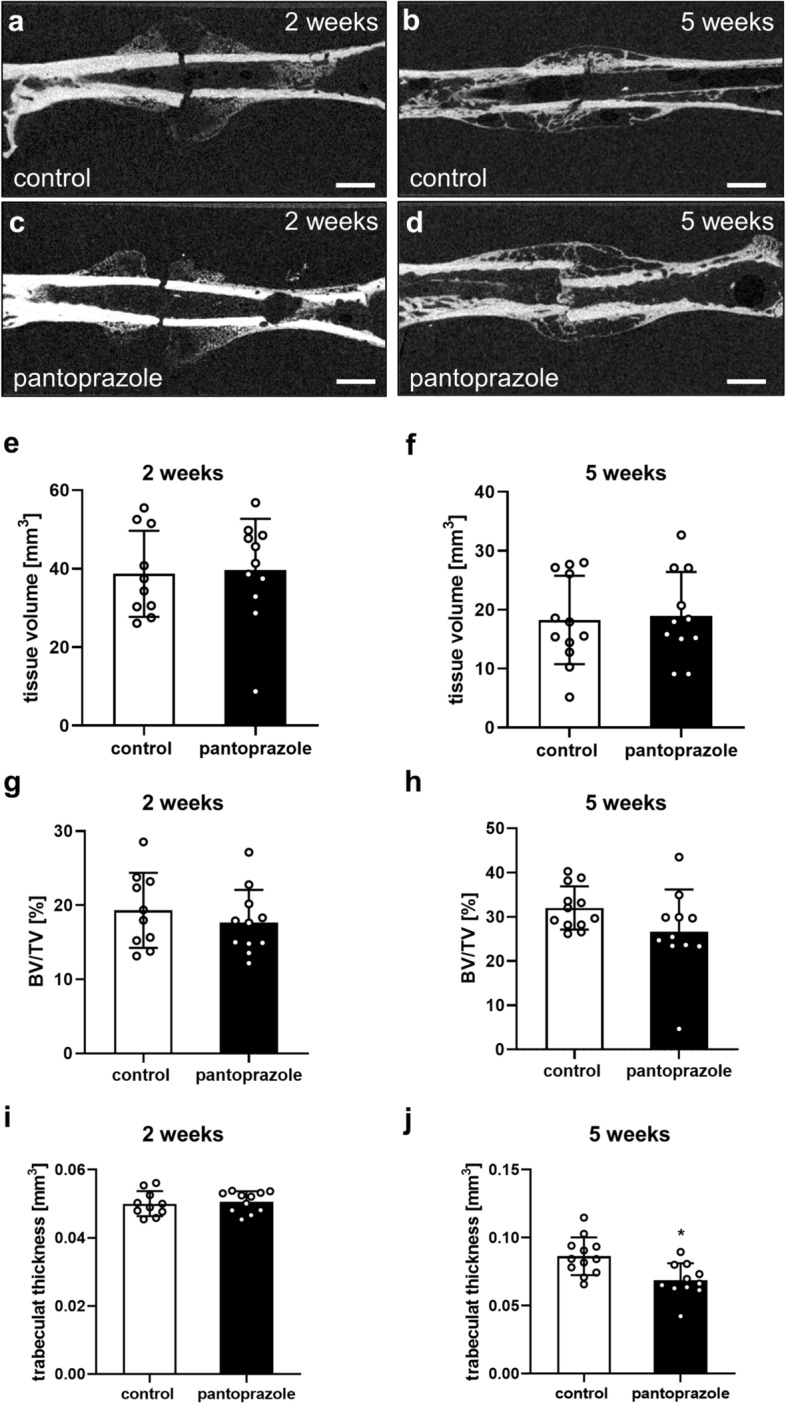
µ-CT analysis of mice femora. Representative µCT images at 2 weeks (**a**,**c**) and 5 weeks (**b**,**d**) after fracture healing in controls (**a**,**b**) and pantoprazole-treated animals (**c**,**d**). Scale bars: 2 mm. (**e**–**j**) µCT analysis of the tissue volume [mm^3^] (**e**, **f**), bone volume/tissue volume (*BV/TV*, [%]) (**g**,**h**) and trabecular thickness [mm] (**i**,**j**) at 2 weeks (**e**,**g**,**i**) and 5 weeks (**f**,**h**,**j**) after fracture healing in controls (*white bars*) and pantoprazole-treated animals (black bars). Means ± SD; n = 10–12; *p < 0.05 vs. control.

The influence of pantoprazole on fracture healing was additionally assessed using µCT analysis. Two weeks after surgery the µCT analysis revealed the formation of a large fracture callus in both controls and pantoprazole-treated animals (Fig. 6a,c). Five weeks after surgery all mice of the two groups showed a complete osseous bridging of the fracture gap (Fig. 6b,d). At 2 (*p* = 0.853) and 5 weeks (*p* = 0.831) after fracture healing the overall tissue volume in pantoprazole-treated animals did not differ from that of controls (Fig. 6e,f). The bone volume to tissue volume ratio was slightly lower after 2 (*p* = 0.438) and 5 weeks (*p* = 0.103) of pantoprazole treatment when compared to controls. However, this difference did not prove to be statistically significant (Fig. 6g,h). While the trabecular thickness of the callus was not affected at 2 weeks (*p* = 0.694) after pantoprazole treatment, the analysis after 5 weeks (*p* = 0.004) of fracture healing revealed a significantly reduced trabecular thickness in pantoprazole-treated animals when compared to controls (Fig. 6i,j).

### Western blot analysis

**Figure 7:**
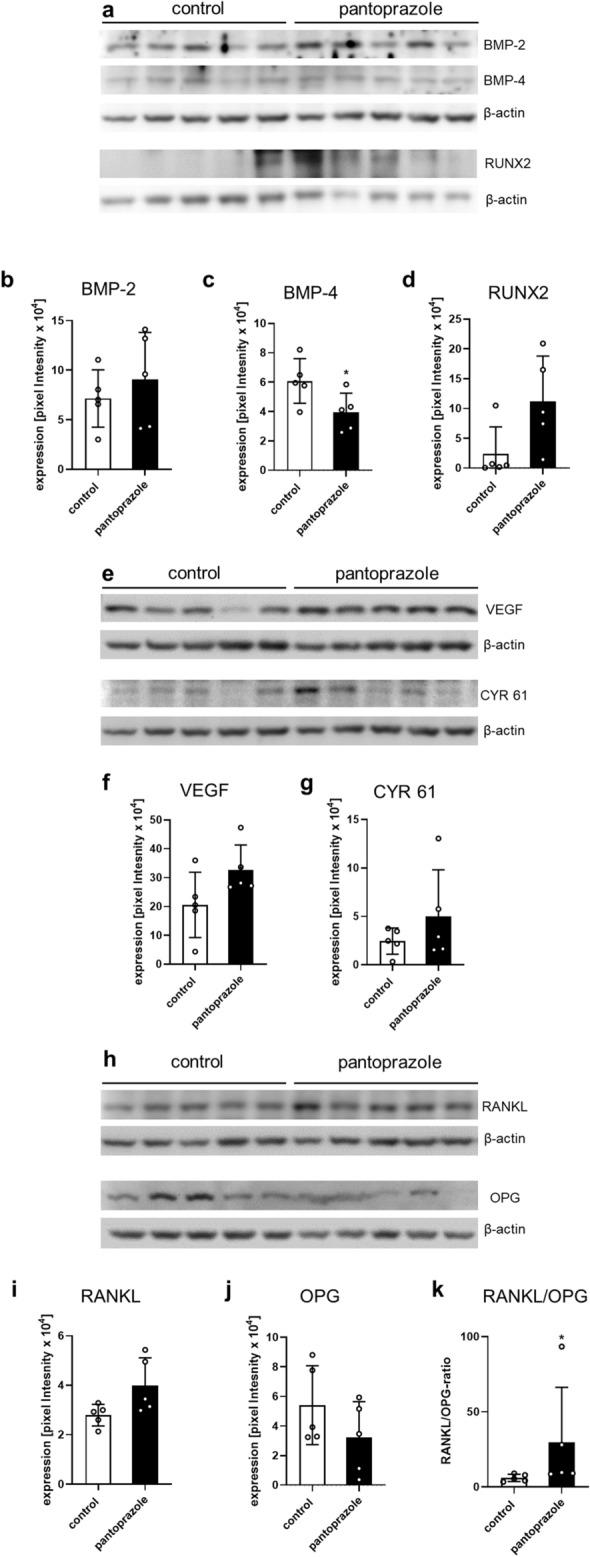
Western blot analysis of mice femora (1). Western blot analysis of the expression of the bone formation markers BMP-2 (**a**,**b**), BMP-4 (**a**,**c**) and RUNX2 (**a**,**d**), the pro-angiogenic factors VEGF (**e**,**f**) and CYR 61 (**e**,**g**), the osteoclastogenesis stimulator RANKL (**h**,**i**) and the osteoclastogenesis inhibitor OPG (**h**,**j**) in the callus of controls (white bars) and pantoprazole-treated animals (black bars) at 2 weeks after fracture healing. (**k**) displays the RANKL/OPG-ratio in the callus of controls (white bars) and pantoprazole-treated animals (black bars). Means ± SD; n = 5; *p < 0.05 vs. control.

At 2 weeks after fracture healing Western blot analysis of the callus tissue demonstrated that pantoprazole treatment significantly reduced the expression of the bone formation marker bone morphogenetic protein (BMP)-4 when compared to controls (Fig. 7a,c) (*p* = 0.045). In contrast, the expression of BMP-2 was not affected in pantoprazole-treated animals (Fig. 7a,b) (*p* = 0.459).

The expression of runt-related transcription factor 2 (RUNX2), an important regulator of osteoblastogenesis, was only slightly but not significantly increased in pantoprazole-treated animals when compared to controls (Fig. 7a,d).

The expression of the pro-angiogenic factors vascular endothelial growth factor (VEGF) (*p* = 0.095) and cysteine-rich protein (CYR) 61 (*p* = 0.690) was almost twofold higher in pantoprazole-treated animals, however, this difference did not prove to be statistically significant (Fig. 7e–g). As a result, we found in pantoprazole-treated animals slightly lower ratios of BMP-2/VEGF (*p* = 0.162) and BMP-2/Cyr 61 (*p* = 0.310), but markedly lower ratios of BMP-4/VEGF (*p* = 0.016) and BMP-4/Cyr 61 (*p* = 0.095) (Table 1).

**Table 1:** Ratio of osteogenic to angiogenic growth factor expression within the callus tissue of controls and pantoprazole-treated animals at 2 weeks after fracture healing.

Osteogenic/angiogenic growth factor expression	Control	Pantoprazole
BMP-2/VEGF	0.42 ± 0.08	0.27 ± 0.05
BMP-2/Cyr 61	4.13 ± 1.31	2.42 ± 0.38
BMP-4/VEGF	0.41 ± 0.13	0.12 ± 0.02*
BMP-4/Cyr 61	4.26 ± 2.0	1.32 ± 0.38

All data are mean ± SEM; *p < 0.05 vs. control.

Moreover, the expression of receptor activator of NF-κB ligand (RANKL), a stimulator of osteoclastogenesis, was increased in pantoprazole-treated animals (Fig. 7h,i) (*p* = 0.056). On the other hand, osteoprotegerin (OPG), an inhibitor of osteoclastogenesis, was decreased after pantoprazole treatment (Fig. 7h,j) (*p* = 0.121). As a result, the RANKL/OPG ratio was significantly higher in pantoprazole-treated animals when compared to controls (Fig. 7k) (*p* = 0.016).

**Figure 8:**
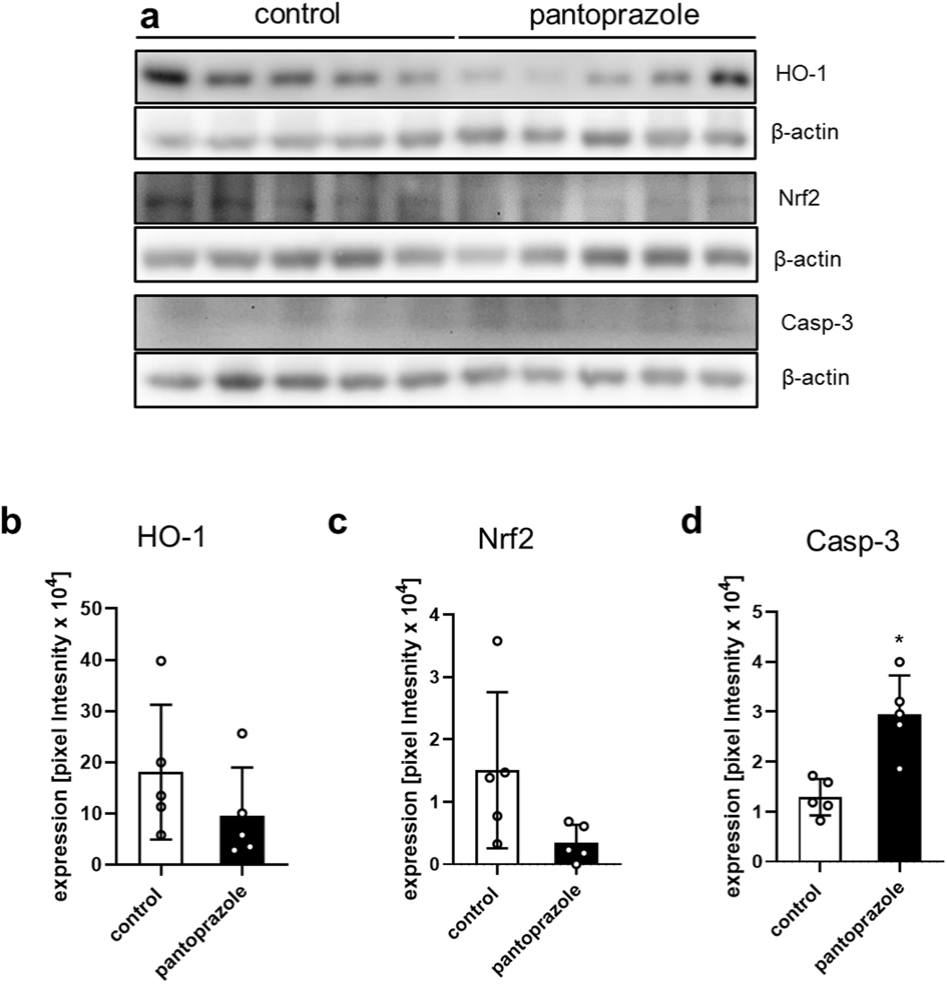
Western blot analysis of mice femora (2). Western blot analysis of the anti-oxidant factors HO-1 (**a**,**b**) and Nrf2 (**a**,**c**) and the pro-apoptotic marker Casp-3 (**a**,**d**) in the callus of controls (white bars) and pantoprazole-treated animals (black bars) at 2 weeks after fracture healing. Means ± SD; n = 5; *p < 0.05 vs. control.

Additional Western blot analyses revealed a reduced expression of heme oxygenase (HO)-1 (Fig. 8a,b) (p = 0.222) and the nuclear factor erythroid 2-related factor 2 (Nrf2) (Fig. 8a,c) (p = 0.077) in pantoprazole-treated animals. In addition, we found a significantly increased expression of the apoptotic marker cleaved caspase-3 (Casp-3) (Fig. 8a,d) (p = 0.002).

## Discussion

The aim of the present study was to analyze the effect of pantoprazole treatment on bone healing in aged mice. Our results demonstrate that pantoprazole impairs fracture repair in aged mice, as indicated by a decreased bone formation, resulting in a reduced biomechanical stiffness. Of interest, the impaired fracture repair was associated with a lower expression of the bone formation marker BMP-4, and a disturbed ratio of pro-angiogenic proteins to osteogenic growth factors.

PPIs are among the most widely used pharmaceuticals in modern medicine. They work by inhibiting the H^+^/K^+^-ATPase enzyme, and, as a result, by elevating the intragastric pH. Therefore, they are used in the management of acid-related gastrointestinal disorders and in the prevention of drug-related side effects on the gastrointestinal tract. However, recent studies emphasize their side effects, including an increased risk of fractures especially among patients with the age of 50 or older^28^. Interestingly, Prause et al.^29^ could show that PPI treatment of osteoclasts in vitro decreases their viability and reduces their TRAP activity, resulting in an overall inhibition of their bone degrading and resorption function. Furthermore, another study performed in vivo revealed that gastrointestinal PPIs delay osteoclastic resorption of bone and calcium phosphate biomaterials^30^. Of note, osteoclasts play a vital role in the process of bone remodeling and fracture repair^31,32^ and their inhibition is associated with a delay of bone healing^33^. In line with these findings, our biomechanical, histological and µCT analyses demonstrate a decreased bone tissue formation and trabecular thickness as well as a reduced bending stiffness in pantoprazole-treated aged animals. In addition, the histological staining revealed a significantly increased number of TRAP-positive osteoclasts 2 weeks after fracture healing. At the same time, Western blot analyses revealed a significantly increased RANKL/OPG ratio in pantoprazole-treated animals. These findings indicate an early accelerated bone turnover after pantoprazole treatment.

There is accumulating evidence that the acid suppressive action of PPIs may lead to a malabsorption of several nutrients, including calcium^34^. Furthermore, hypocalcemia leads to an endocrine feedback loop stimulating parathyroid hormone (PTH) production and secretion. PTH enhances osteoclastic bone resorption and liberates both calcium and phosphate from the skeleton^35^. In a recent study, Fischer et al.^36^ demonstrated that calcium and vitamin D deficiency in mice with ovariectomy-induced osteoporosis leads to increased serum levels of PTH and osteoclast activity. Thus, it may be speculated that the increased osteoclast activity, observed in our present study, is caused by a pantoprazole-induced malabsorption of calcium, which subsequently may have led to increased PTH. This early dominant osteoclastic activity may inhibit adequate bone formation during the process of healing, possibly contributing to the increased fragility of the bone and the reduced healing quality.

Of interest, in the aged mice of the present study pantoprazole suppressed the expression of the bone formation marker BMP-4, whereas the expression of BMP-2 was not affected. BMP-2 and BMP-4 were analyzed, because they belong to the best characterized and most osteoinductive members of the BMP family, displaying a biological activity throughout different stages of bone healing^37,38^. Because BMP-4 expression was suppressed in pantoprazole-treated mice at 2 weeks after fracture healing, we propose that pantoprazole affects bone metabolism through the regulation of bone formation. The BMP action in fracture healing includes the conversion of soft callus into bone, involving the resorption of calcified cartilage and the formation of new bone tissue^39^. Thus, the lower expression of bone formation markers, i.e. BMP-4, observed after pantoprazole treatment, may have resulted in a delayed fracture healing with a lower callus stability and, thus, a reduced bending stiffness.

Vascularization is thought to be a prerequisite for successful bone regeneration. VEGF and CYR 61 are recognized as major pro-angiogenic factors, which also exert osteogenic activities, improving the process of bone formation and accelerating fracture repair in experimental animal models^40,41^. This is in contrast with the results of the present study. In fact, we found an increased expression of VEGF and CYR 61 in the callus tissue of the pantoprazole-treated animals, which, however, was associated with a reduced bone formation and a lower bending stiffness. Of interest, Peng et al.^42,43^ demonstrated that VEGF alone is not capable of initiating the cascade of bone formation and that overexpression of VEGF can even impair the process of bone regeneration and healing. In line with this view, Garcia et al.^44^ found in a murine femoral fracture model an increased VEGF expression in the fibrous callus tissue of nonunions when compared with the osseous callus tissue of successfully healed fractures. Accordingly, Weiss et al.^45^ and Sarahrudi et al.^46^ could show that serum levels of VEGF are increased in patients with nonunions when compared with that of patients with normal healing fractures. However, it remains to be determined, whether a massive overexpression of angiogenic growth factors such as VEGF and CYR 61 causes nonunion formation or whether a nonunion formation caused by other factors induces a compensatory overexpression of these factors.

Moreover, it could be demonstrated that impaired fracture healing in the elderly is associated with a dysfunction in the bone vascular system, resulting in a delayed angiogenesis and a decreased vascularization during fracture repair^2,47^. In fact, at early healing time points during healing fracture callus of young mice presents with a significantly higher surface density of blood vessels when compared with that of aged mice^48^. The impaired healing process caused by pantoprazole treatment may aggravate the dysfunction of the vascular system. In line with this hypothesis we found a significantly decreased vessel density within the callus tissue of pantoprazole-treated animals at 2 weeks after facture healing. This may cause hypoxic conditions in the fracture callus, which results in an increased expression of the pro-angiogenic growth factors VEGF and CYR 61. In contrast, growth factors for osteoblastic differentiation like BMP-4 and new bone formation are down-regulated, because in this critical setting of hypoxia cell survival at the fracture site is of principal importance. This view is in line with the results of the study of Garcia et al.^44^, who found an overexpression of VEGF when compared to BMP-2 and BMP-4 in nonunion formation, which is most likely caused by an increased hypoxic environment and impaired functional vascularization in segmental bone defects^44^. Finally, Peng et al.^43^ reported that excessive expression of VEGF in relation to BMP-4 impairs fracture healing by a differentiation of mesenchymal stem cells toward an endothelial lineage rather than a differentiation into osteoblastic cells.

Interestingly, the reduction of the bone formation marker BMP-4 by pantoprazole is in line with the results of our previous study, in which we analyzed the effects of pantoprazole on fracture healing in young adult animals^18^. Nonetheless, there are some major differences in response when comparing the effects of pantoprazole on fracture healing in aged mice compared to young adult animals. In pantoprazole-treated aged mice bending stiffness at 2 and 5 weeks is ~ 50% lower compared to pantoprazole-treated young adult mice^18^. However, this difference is most probably caused by aging, because in non-treated aged mice bending stiffness at 2 and 5 weeks is also ~ 50% lower compared to non-treated young adult mice^18^. However, in aged mice pantoprazole provoked an early deterioration of fracture healing, as shown by a markedly reduced ratio of osseous tissue within the callus at 2 weeks after fracture healing. In contrast, in young adult animals pantoprazole affected the ratio of osseous tissue only during the later course at 5 weeks of fracture healing^18^. Most importantly, in pantoprazole-treated young adult animals RANKL, a stimulator of osteoclastogenesis, was found reduced, while in pantoprazole-treated aged mice the number of TRAP-positive cells and the expression of RANKL was found markedly increased during the initial period of healing and reduced only during the late time course at 5 weeks. These latter findings indicate a different mode of action of pantoprazole in aged compared to young adult mice. In aged animals, pantoprazole exerts impaired healing due to an early, accelerated and overwhelming osteoclastic response, most likely caused by enhanced PTH serum levels due to calcium malabsorption. In contrast, in young adult animals, pantoprazole exerts impaired healing due to a reduced osteoclast activity with delayed bone remodeling, which is discussed to be caused by an inhibition of the osteoclastic V-ATPase^18^.

The process of aging is associated with substantial changes in gene expression and metabolic control, as well as the production of high levels of reactive oxygen species (ROS)^49^. These toxic radicals are thought to be crucially involved in cellular senescence and induce cell injury by damaging nuclear acids and proteins^50^. Furthermore, ROS are produced during the initial phase of fracture healing under ischemic and inflammatory conditions^50^. The damage induced by ROS is attenuated by antioxidant enzymes generated during fracture healing. These enzymes are capable of neutralizing free radicals before they can harm cellular components^50^. Interestingly, we found a reduced expression of HO-1 and Nrf2 within the callus tissue of pantoprazole-treated animals. These two molecules are widely accepted to play an essential role in the protection against oxidative stress and apoptotic injury^51^. Accordingly, we found an increased expression of the apoptotic marker Casp-3 within the callus tissue of pantoprazole-treated animals, indicating enhanced cell and tissue damage. Therefore, pantoprazole may impair the process of fracture healing due to the increased amount of ROS-induced injury.

The present study has some limitations. In order to fully exploit the effects of pantoprazole on fracture healing, we have used in the present study a higher pantoprazole dose when compared to that used in clinical practice. Therefore, the effect of pantoprazole on bone regeneration in patients may be less pronounced when compared to that observed in the present study. Hence, clinical trials are necessary to confirm the results of this experimental study. In addition, despite the evidence of negative effects of pantoprazole on bone healing, the prevention of peptic ulcers and reflux esophagitis is always of major importance, especially in elderly individuals. Thus, although the results of the present study suggest that pantoprazole should be used with caution during fracture healing in the elderly, it has always to be carefully weighed up whether PPI treatment should be stopped.

In conclusion, the present study demonstrates that pantoprazole treatment in aged mice impairs fracture healing most probably by a too early stimulation of osteoclast activity, a downregulation of BMP-4 and antioxidant factors, as well as a disturbed ratio of angiogenic to osteogenic growth factors.

## Methods

### Animals and specimens

For the present study a total number of 54 CD-1 mice of both sexes with an age of 16–18 months were used. The animals were bred at the Institute for Clinical and Experimental Surgery, Saarland University. The experiments were approved by the local governmental animal protection committee (Landesamt für Verbraucherschutz, Abteilung C Lebensmittel- und Veterinärwesen, Saarbrücken, Germany) and were conducted in accordance with the European legislation on protection of animals (Guide line 2016/63/EU) and the National Institutes of Health Guidelines for the Care and Use of Laboratory Animals (http:// oacu.od.nih.gov/regs/index.htm. Eighth Edition; 2011). Twenty-two mice were treated daily by intraperitoneal (i.p.) injection of 100 mg/kg body weight (BW) pantoprazole (Nycomed, Konstanz, Germany). Another 22 mice treated with vehicle (saline) served as controls. The dose of pantoprazole chosen in the present study was the same as was given in the previous study with young adult animals^18^ and corresponds to doses used in various experimental studies^52–54^.

### Surgical procedure

Mice were anesthetized by i.p. injection of xylazine (12 mg/kg BW) and ketamine (90 mg/kg BW). Femoral fractures were induced as described previously in detail^18^. Under aseptic conditions a medial parapatellar incision was performed at the right knee and the patella was dislocated laterally. After drilling a hole (diameter of 0.5 mm) into the intercondylar notch an injection needle with a diameter of 0.4 mm was placed into the intramedullary canal. Subsequently, a tungsten guidewire (diameter of 0.2 mm) was inserted through the needle. After removal of the needle, the femur was fractured by a 3-point bending device and an intramedullary medical stainless steel screw (length of 17.2 mm, diameter of 0.5 mm; AO Foundation, Research Implants System, Davos, Switzerland) was implanted over the guidewire to stabilize the fracture^55^. After fixation of the fracture the wound was closed using 5-0 synthetic sutures. Adequate reduction of the fracture and position of the implant were confirmed by radiography (MX-20, Faxitron X-ray Corporation, Wheelin, IL, USA). All fractures were simple, transverse midshaft fractures according to the AO classification type A2 fracture. In none of the animals a comminuted or incomplete fracture was observed. For analgesia the mice received tramadol-hydrochloride (Grünenthal, Aachen, Germany) in the drinking water (1 mg/ml) from day 1 before surgery until day 7 after surgery^56^.

### Radiological analysis

At the end of the observation period the animals were re-anesthetized and lateral X-rays (MX-20, Faxitron X-ray Corporation) of the healing femora were performed. Fracture healing was analyzed according to the classification of Goldberg with stage 0 indicating radiological nonunion, stage 1 indicating possible union and stage 2 indicating radiological union^57,58^.

### Biomechanical analysis

For biochemical analysis, femora were resected at 2 and 5 weeks after fracture and freed from soft tissue. After removal of the implants callus stiffness was measured using a three-point bending device (Mini-Zwick Z 2.5; Zwick, Ulm, Germany). Due to the different time points of healing studied, the loads which had to be applied varied markedly between the individual animals. Loading was stopped individually in every case when the actual load–displacement curve deviated more than 1% from linearity^59^. To guarantee standardized measuring conditions, femora were always mounted with the ventral aspect upwards. A working gauge length of 6 mm was used. Applying a gradually increasing bending force with 1 mm/min, the bending stiffness (N/mm) was calculated from the linear elastic part of the load displacement diagram^56^. The application of only a non-destructive force was controlled macroscopically and, later on, microscopically during the histological analysis. To account for differences in bone stiffness of the individual animals, the unfractured left femora were also analyzed, serving as internal control. All values of the fractured femora are given as absolute values and, additionally, in percent of the corresponding unfractured femora.

### Histomorphometric analysis

For histological analysis, femora were analyzed at 2 and 5 weeks after fracture healing. The bones were fixed in IHC zinc fixative (BD Pharmingen, San Diego, CA) for 24 h, decalcified in 13% EDTA solution for 2 weeks and then embedded in paraffin. Longitudinal sections of 5 µm thickness were stained with Safranin-O. At a magnification of × 1.25 (BX60, Olympus, Tokyo, Japan; Axio Cam and Axio Vision 3.1, Carl Zeiss, Oberkochen, Germany) structural indices were calculated according to the suggestion provided by Gerstenfeld et al.^60^ using the ImageJ Analysis System (NIH, Bethesda, MD, USA). These included total callus area (bone, cartilaginous and fibrous callus area)/femoral bone diameter (cortical width plus marrow diameter) at the fracture gap (CAr/BDm [mm]), bone (total osseous tissue) callus area/total callus area (TOTAr/CAr [%]), cartilaginous callus area/total callus area (CgAr/CAr [%]), and fibrous tissue callus area/total callus area (FTAr/CAr [%])^18,56^.

Additionally, tartrate-resistant acid phosphatase (TRAP) activity was analyzed in the callus at 2 and 5 weeks after fracture healing. Therefore, bones were fixed in IHC zinc fixative for 24 h, decalcified in 13% EDTA solution for 2 weeks and then embedded in paraffin. After deparaffinizing again, longitudinal sections of 5 µm thickness were incubated in a mixture of 5 mg naphthol AS-MX phosphate and 11 mg fast red TR salt in 10 ml 0.2 M sodium acetate buffer (pH 5.0) for 1 h at 37 °C. Sections were counterstained with methyl green and covered with glycerine gelatine. TRAP-positive multinucleated cells (three or more nuclei each cell) were counted as described before in detail^18^. The data on histomorphological osteoclast analyses are given as numbers of osteoclasts per high-power field (HPF). The osteoclast analysis differentiated between the number of osteoclasts within bone tissue and the number of osteoclasts within cartilaginous tissue^18^.

Moreover, the number of microvessels was analyzed in the callus at 2 and 5 weeks after fracture healing. Sections were additionally cut and stained with hematoxylin and eosin (HE) according to standard procedures. The presentation of a lumen with a typical vascular wall morphology was necessary to classify the identified structure as a microvessel. The number of microvessels in controls and pantoprazole-treated animals was counted at a magnification of × 400 (Olympus BX60 microscope) in the central part of the periosteal callus in 4 high power fields (HPF) per specimen [vessels/HPF]^61^.

In addition, the number of osteoblasts was analyzed in the callus at 2 and 5 weeks after fracture healing. Sections were additionally cut and stained with an anti-osteocalcin antibody (Abcam, Cambridge, UK), an osteoblast marker, which was detected by its corresponding secondary antibody. Osteocalcin-positive osteoblasts were counted in the central part of the periosteal callus in 4 high power fields (HPF) per specimen [osteocalcin-positive osteoblasts/HPF].

### µ-CT analysis

The femora were scanned (Skyscan 1172, Bruker, Billerica, MA) at 2 and 5 weeks after fracture at a spatial resolution of 8.9 mm with a standardized setup, as described previously^62^. Images were stored in three-dimensional (3D) arrays. To express gray values as mineral content (bone mineral density; BMD), calcium hydroxyapatite (CaHA) phantom rods with known BMD values (0.250 g and 0.750 g [CaHA/cm^3^]) were used for calibration. The ROI was contoured manually on each transversal slide, including exclusively newly formed callus tissue but excluding the original cortical bone. For the analysis both periosteal and intramedullary callus tissue was included. The ROI was interpolated between each transversal slide by a CT analyzer software (CTAnalyser, Bruker, Billerica, MA, USA) to guarantee the inclusion of the complete callus tissue. For each specimen the tissue volume (TV, [mm^3^]), the bone volume fraction of the tissue volume (BV/TV, [mm^3^]) and the trabecular thickness [mm] from callus ROI was determined.

### Western blot analysis

Protein expression within the callus tissue was determined by Western blot analysis, including the expression of bone morphogenetic protein-2 (BMP-2) and -4 (BMP-4), the vascular endothelial growth factor (VEGF), cysteine-rich protein (CYR) 61, receptor activator of NF-κB ligand (RANKL) and osteoprotegerin (OPG). The callus tissue was frozen and stored at − 80 °C until required. Analyses were performed from callus tissue at 2 weeks after fracture healing. After saving the whole-protein fraction, analysis was performed using the following antibodies: rabbit anti-mouse BMP-2/BMP-4 (both 1:25, Santa Cruz Biotechnology, Heidelberg, Germany), RUNX2 rabbit anti-mouse (1:50, Abcam, Cambridge, UK), rabbit anti-mouse VEGF (1:100, Santa Cruz Biotechnology), goat anti-mouse CYR 61 (1:100, Santa Cruz Biotechnology), rabbit anti-mouse RANKL (1:25, Abcam) rabbit anti-mouse OPG (1:50, Bioss Antibodies, Woburn, USA), rabbit anti-mouse HO-1 (1:50, Enzo Life Sciences by Biomol GmbH, Homburg), rabbit anti-mouse Nrf-2 (1:50, Cell Signaling Technology, Frankfurt), rabbit anti-mouse Casp-3 (1:50, R&D Systems, Wiesbaden). Primary antibodies were incubated at 4 °C overnight and were followed by corresponding horseradish peroxidase-conjugated secondary antibodies (1.5 h at room temperature, 1:1000, R&D Systems, Minneapolis, USA + DakoCytomation, Hamburg, Germany). Protein expression was visualized by means of luminol-enhanced chemiluminescence after exposure of the membrane to the Intas ECL Chemocam Imager (Intas Science Imaging Instrument GmbH, Göttingen, Germany) and normalized to ß-actin signals (1:5000, mouse anti-mouse ß-actin, Sigma-Aldrich) to correct for unequal loading (Fig. S1).

### Statistics

All data are given as means ± SD. After proving the assumption for normal distribution (Kolmogorov–Smirnov test) and equal variance (F-test), comparison between the two experimental groups was performed by Student’s t-test. For non-parametrical data Mann–Whitney U-test was used. Statistics were performed using SigmaPlot 13.0 software (Systat Software GmbH, Erkrath, Germany). A p-value < 0.05 was considered to indicate significant differences^63^.

## Supplementary Information

Supplementary Information.

## Data Availability

All data generated or analyzed during this study are included in this published article.
