# Supplementary material for: Pantoprazole impairs fracture healing in aged mice
Source: Sci Rep. 2020 Dec 23;10:22376. doi: 10.1038/s41598-020-79605-3 (PMC7758334; doi:10.1038/s41598-020-79605-3)
Supplement: Supplementary file 1 — Supplementary Information. [file 41598_2020_79605_MOESM1_ESM.docx]

**Pantoprazole impairs fracture healing in aged mice**

Maximilian M. Menger^1,2#^, Philipp Bremer^1#^, Claudia Scheuer^1^, Mika F. Rollmann^2^, Benedikt J. Braun^2^, Steven C. Herath^2^, Marcel Orth^3^, Thomas Später^1^, Tim Pohlemann^3^, Michael D. Menger^1^, Tina Histing^1,2^

*^1^Institute for Clinical & Experimental Surgery, Saarland University, 66421 Homburg/Saar, Germany*

*^2^Department of Trauma and Reconstructive Surgery, Eberhard Karls University Tuebingen, BG Trauma Center Tuebingen, 72076 Tuebingen, Germany*

*^3^Department of Trauma, Hand and Reconstructive Surgery, Saarland University, 66421 Homburg/Saar, Germany*

^#^Contributed equally to this work

**^*^Address for correspondence:**

Maximilian M. Menger, M.D.

Institute for Clinical & Experimental Surgery

Saarland University

66421 Homburg/Saar

Germany

phone: +49 6841 16 26550

fax: +49 6841 16 26553

e-mail: maximilian.menger@uks.eu


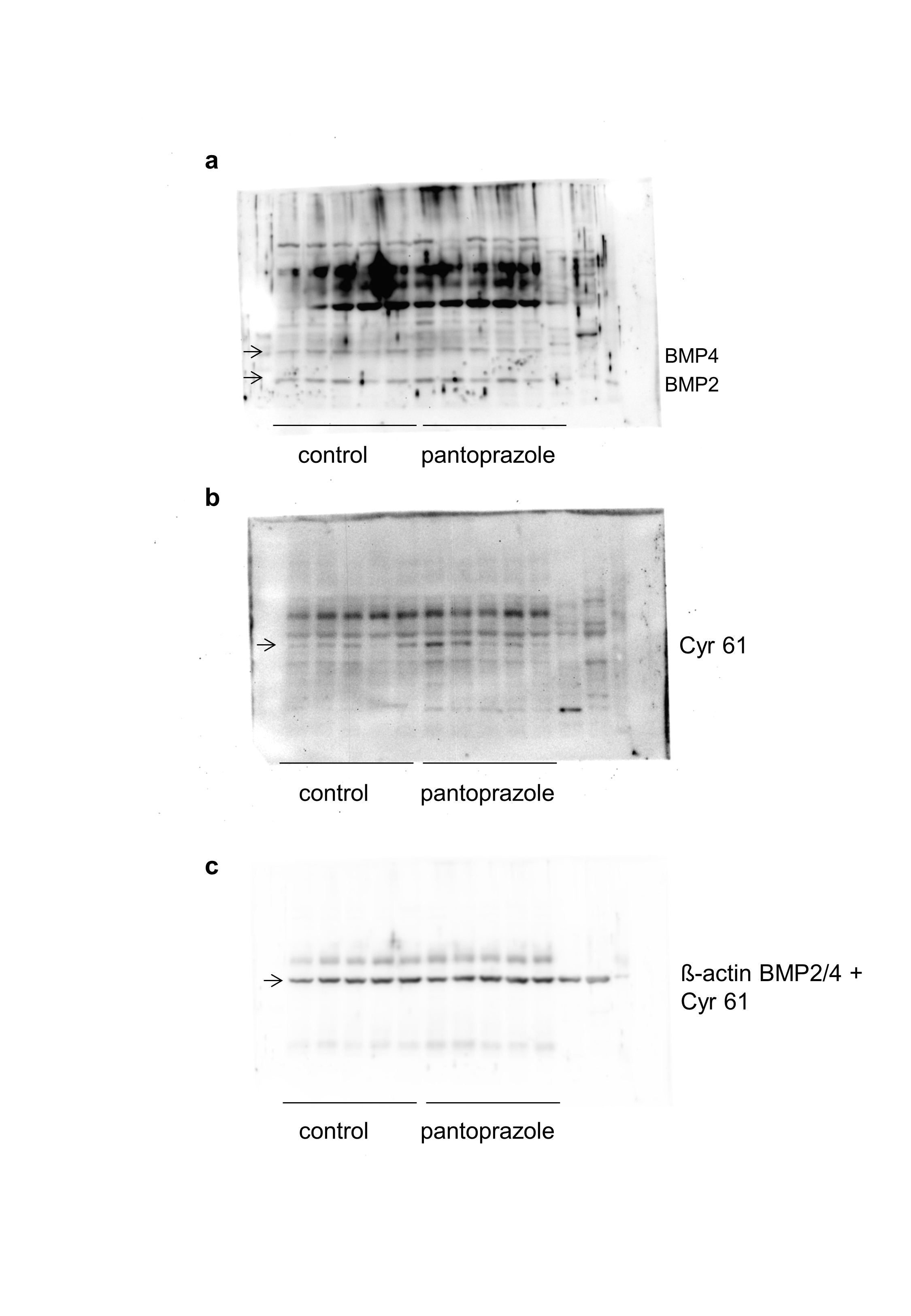


**Figure S1. Full-length Western blots (1).** Full length Western blots of BMP-2, BMP-4 (**a**) and Cyr 61 (**b**) of the callus tissue of controls and pantoprazole-treated animals at 2 weeks after fracture healing. Corresponding β-actin of BMP2/4 and Cyr 61 (**c**) of the callus tissue of controls and pantoprazole-treated animals at 2 weeks after fracture healing. Arrowheads indicate the respective protein.


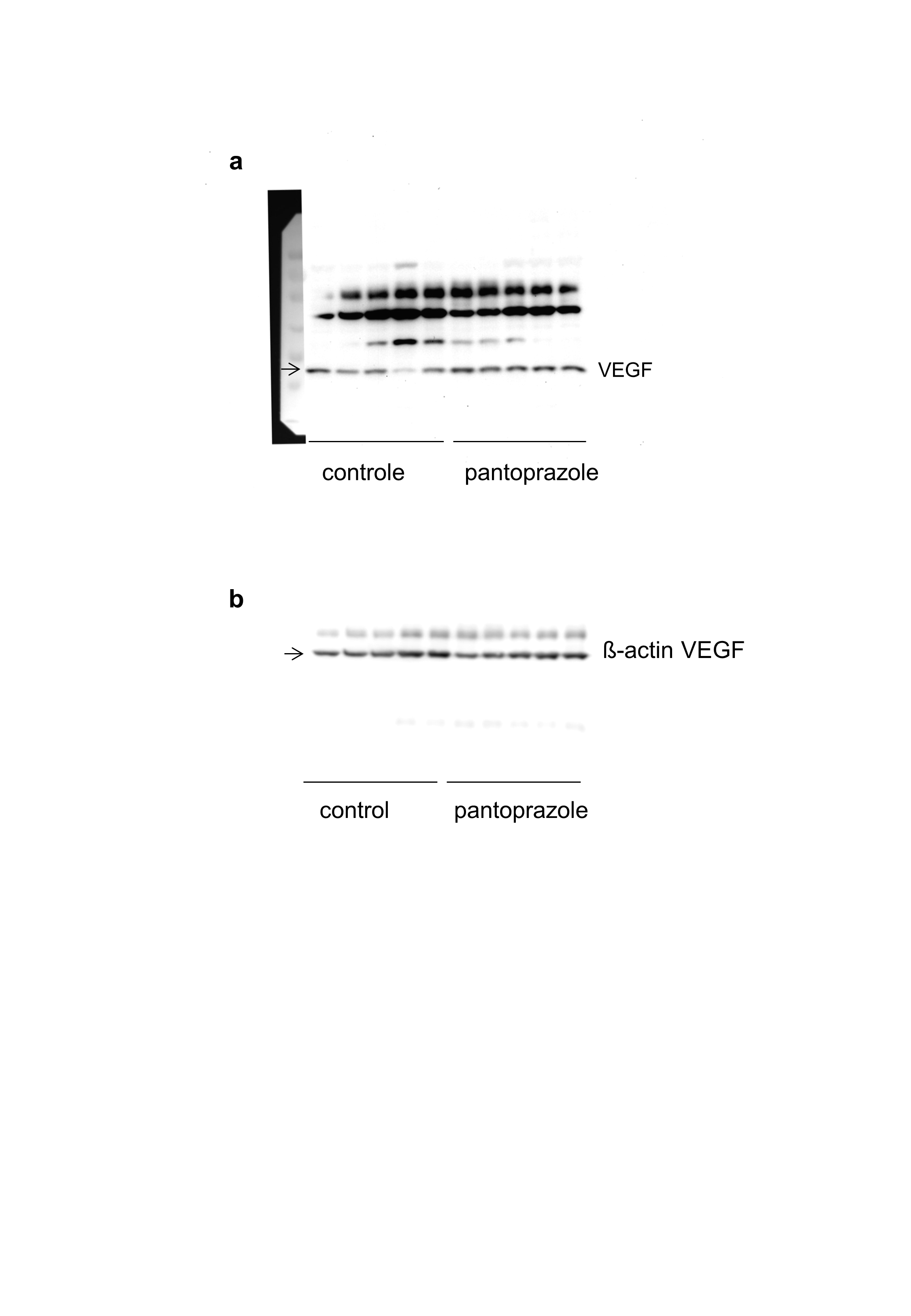


**Figure S2. Full-length Western blots (2).** Full length Western blots of VEGF (**a**) of the callus tissue of controls and pantoprazole-treated animals at 2 weeks after fracture healing. Corresponding β-actin of VEGF (**b**) of the callus tissue of controls and pantoprazole-treated animals at 2 weeks after fracture healing. Arrowheads indicate the respective protein.


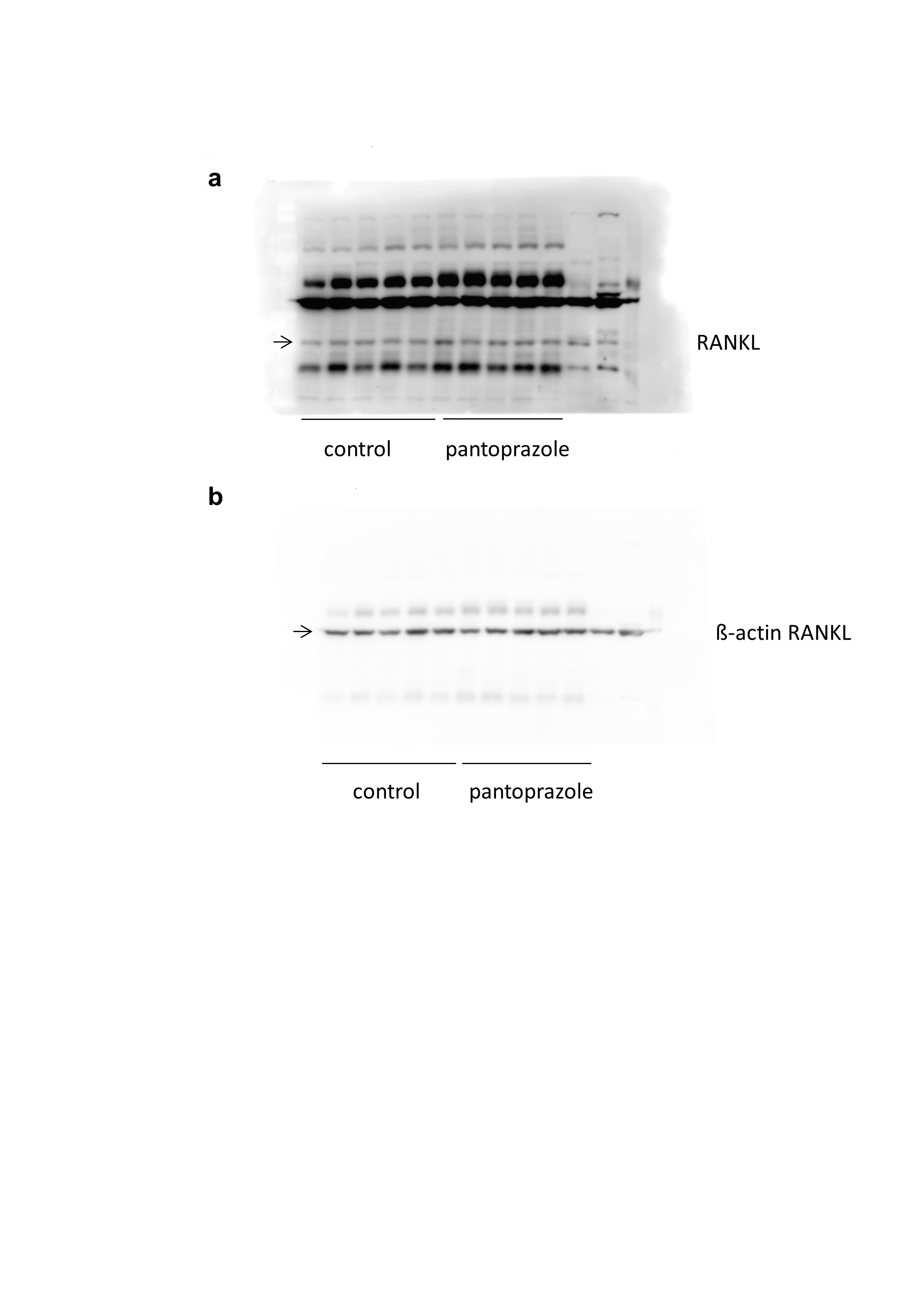


.

**Figure S3. Full-length Western blots (3).** Full length Western blots of RANKL (**a**) of the callus tissue of controls and pantoprazole-treated animals at 2 weeks after fracture healing. Corresponding β-actin of RANKL (**b**) of the callus tissue of controls and pantoprazole-treated animals at 2 weeks after fracture healing. Arrowheads indicate the respective protein.


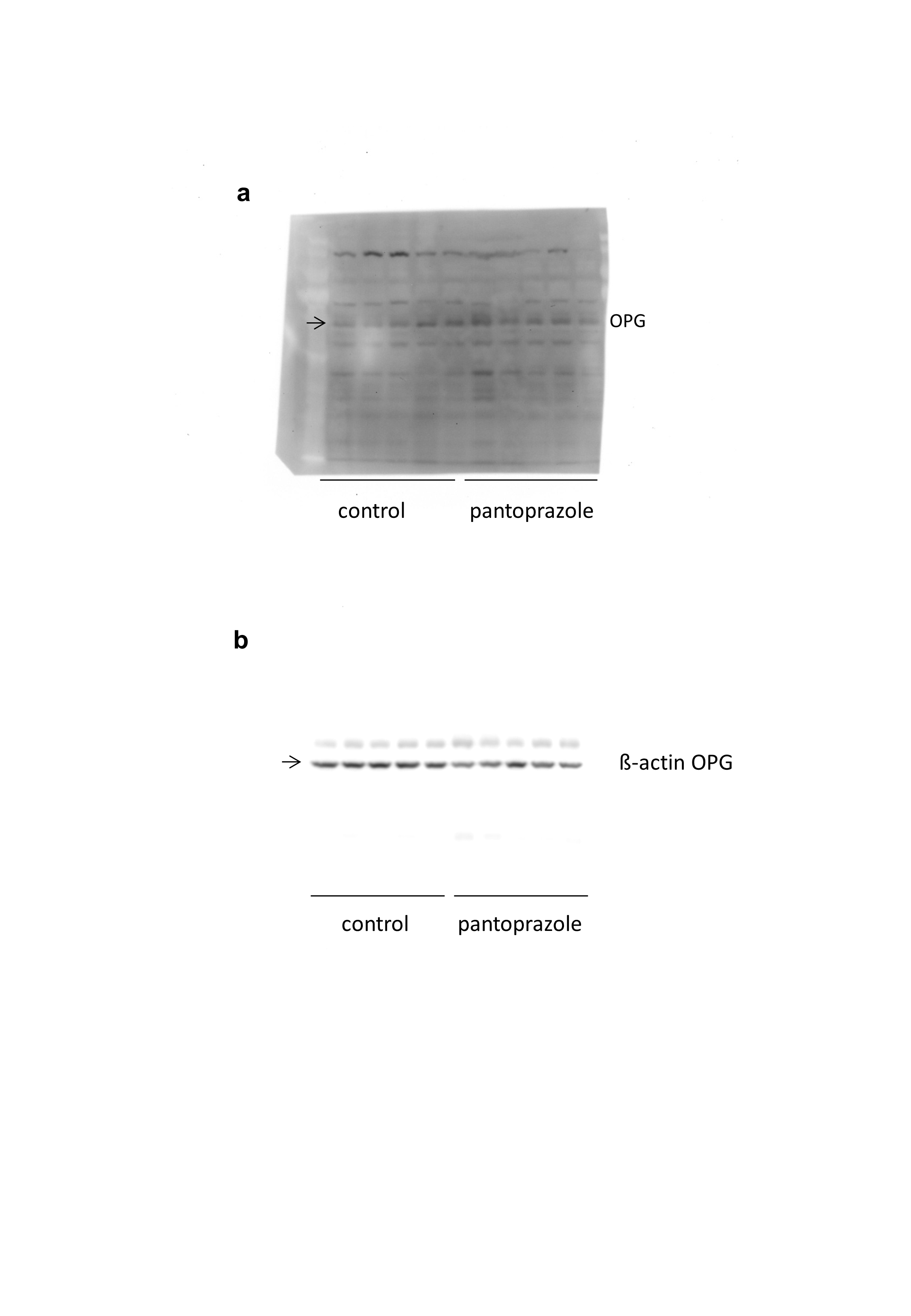


**Figure S4. Full-length Western blots (4).** Full length Western blots of OPG (**a**) of the callus tissue of controls and pantoprazole-treated animals at 2 weeks after fracture healing. Corresponding β-actin of OPG (**b**) of the callus tissue of controls and pantoprazole-treated animals at 2 weeks after fracture healing. Arrowheads indicate the respective protein.


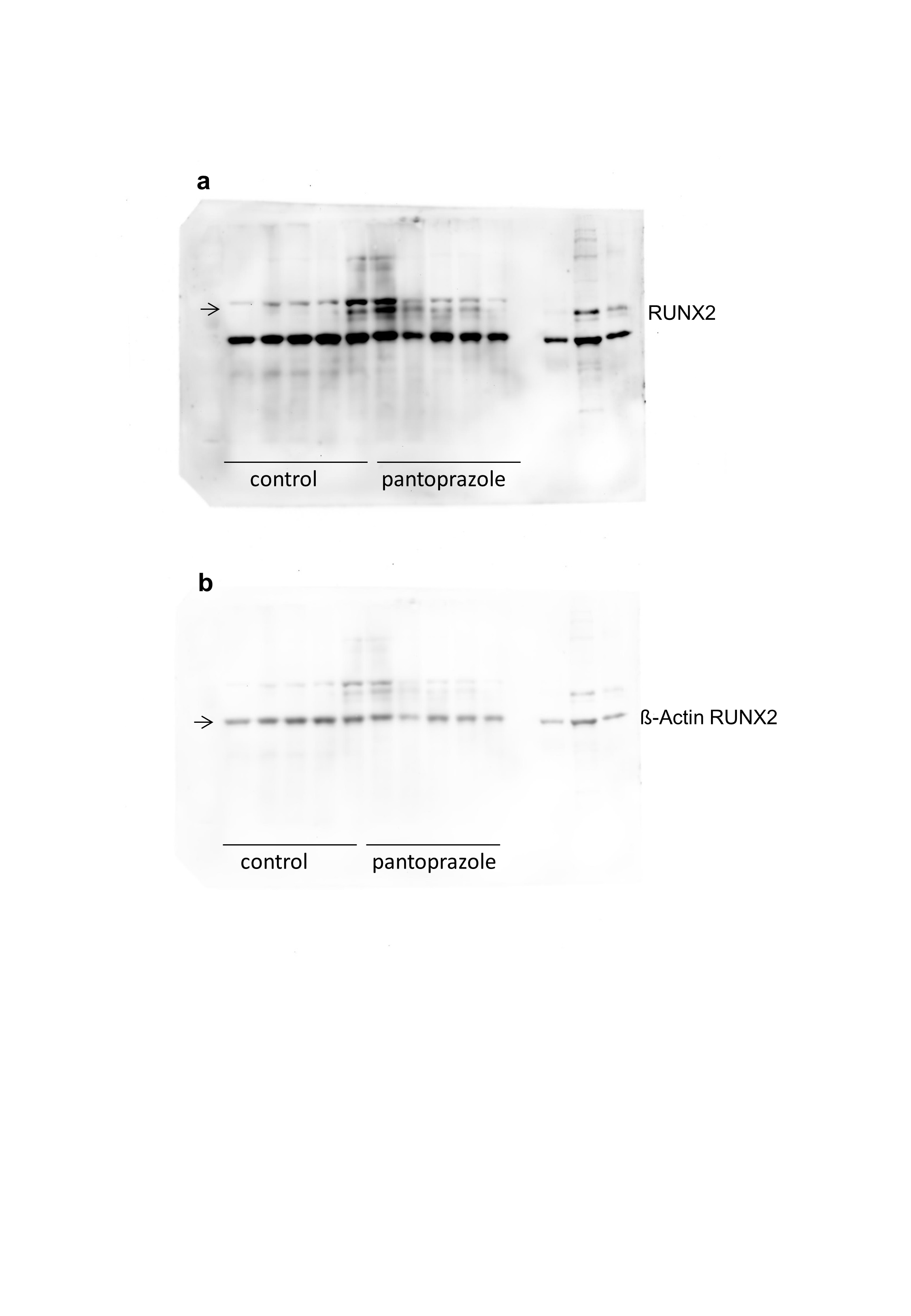


**Figure S5. Full-length Western blots (5).** Full length Western blots of RUNX2 (**a**) of the callus tissue of controls and pantoprazole-treated animals at 2 weeks after fracture healing. Corresponding β-actin of RUNX2 (**b**) of the callus tissue of controls and pantoprazole-treated animals at 2 weeks after fracture healing. Arrowheads indicate the respective protein.


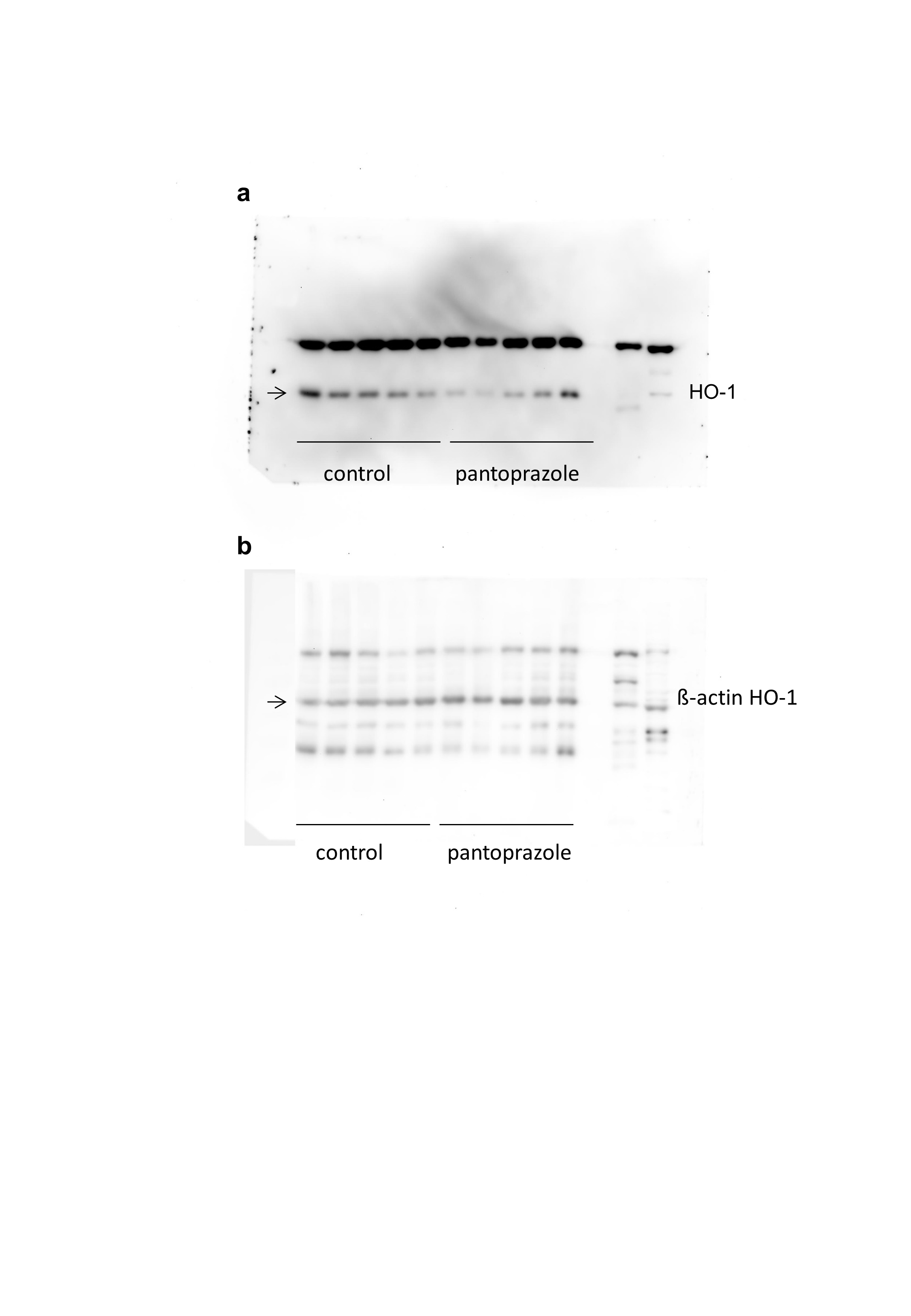


**Figure S6. Full-length Western blots (6).** Full length Western blots of HO-1 (**a**) of the callus tissue of controls and pantoprazole-treated animals at 2 weeks after fracture healing. Corresponding β-actin of HO-1 (**b**) of the callus tissue of controls and pantoprazole-treated animals at 2 weeks after fracture healing. Arrowheads indicate the respective protein.


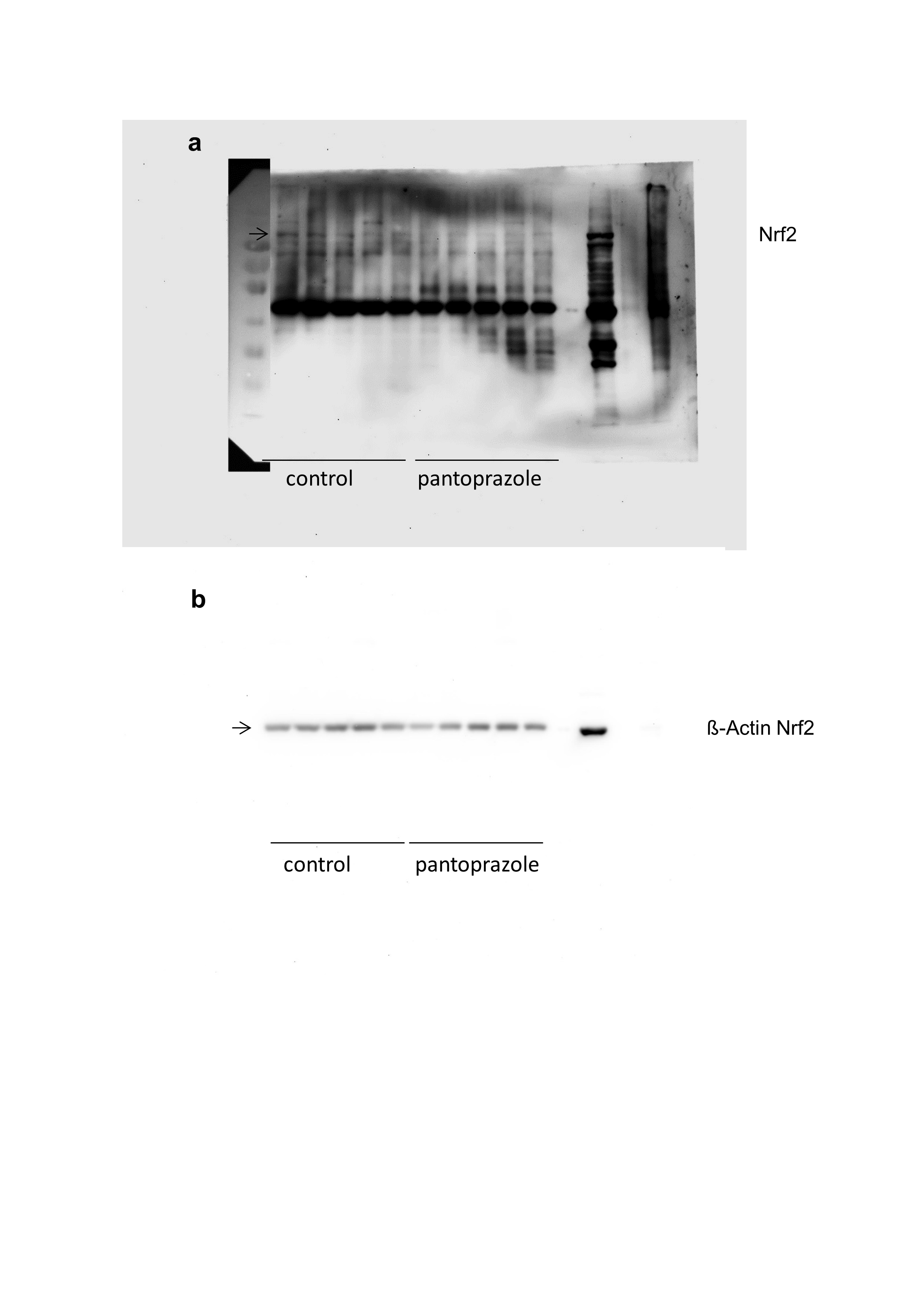


**Figure S7. Full-length Western blots (7).** Full length Western blots of Nrf2 (**a**) of the callus tissue of controls and pantoprazole-treated animals at 2 weeks after fracture healing. Corresponding β-actin of Nrf2 (**b**) of the callus tissue of controls and pantoprazole-treated animals at 2 weeks after fracture healing. Arrowheads indicate the respective protein.


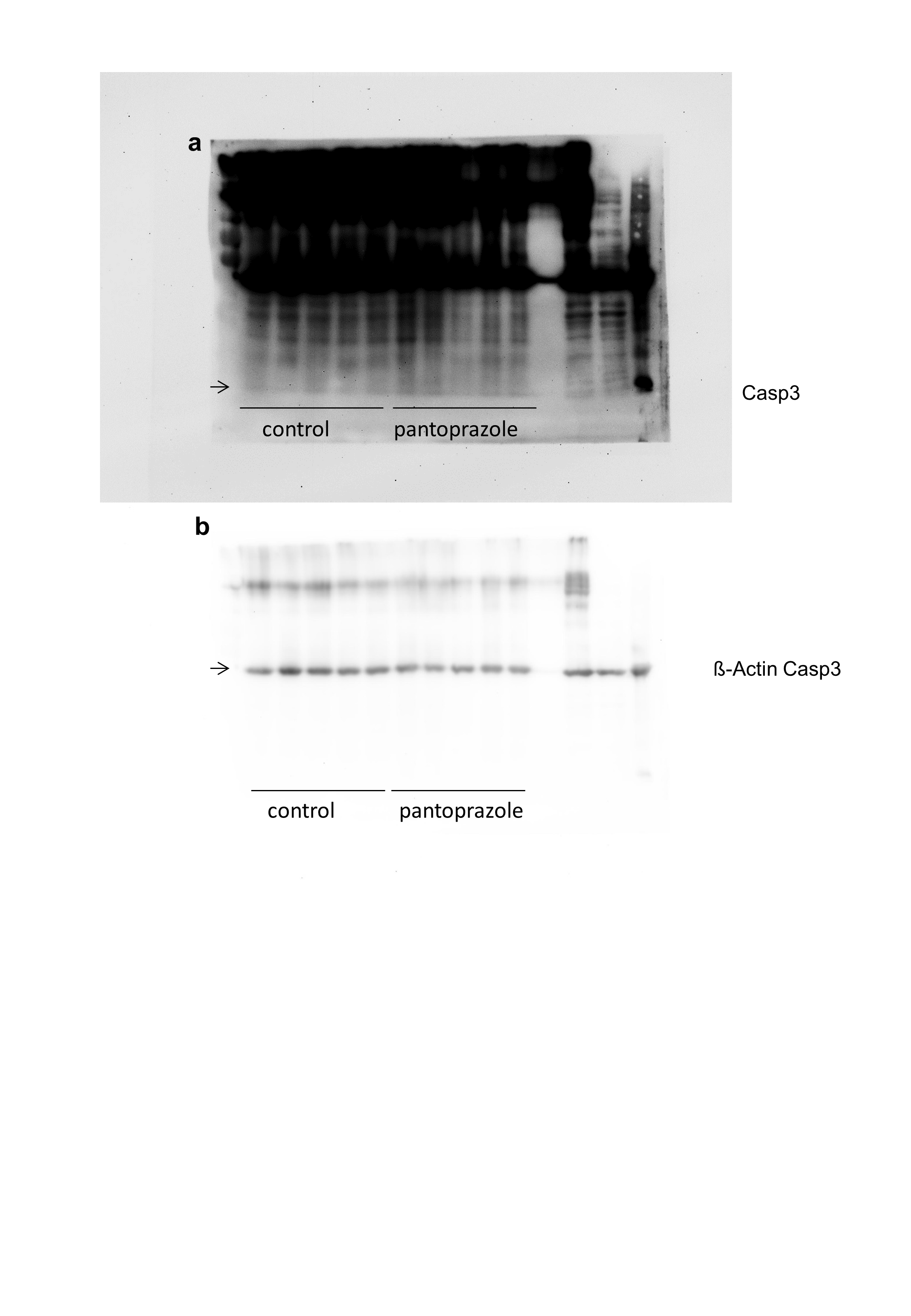


**Figure S8. Full-length Western blots (8).** Full length Western blots of Casp3 (**a**) of the callus tissue of controls and pantoprazole-treated animals at 2 weeks after fracture healing. Corresponding β-actin of Casp3 (**b**) of the callus tissue of controls and pantoprazole-treated animals at 2 weeks after fracture healing. Arrowheads indicate the respective protein.
